# Squamous Cell Carcinoma With Unusual Benign-Appearing Cystic Features on Histology

**DOI:** 10.7759/cureus.33610

**Published:** 2023-01-10

**Authors:** Usman Asad, Suzanne Alkul, Ikue Shimizu, Jennifer Ranario

**Affiliations:** 1 Department of Dermatology, Baylor College of Medicine, Houston, USA

**Keywords:** squamous cell carcinoma, mohs surgery, milia, histopathology, frozen sections, cyst

## Abstract

Squamous cell carcinoma (SCC) is a malignancy that arises from keratinocytes of the epidermis. Cystic variants of several types of cutaneous neoplasms have been rarely described in the literature. We report a case of biopsy-proven, well-differentiated invasive SCC treated with Mohs surgery. On frozen sections, the pathology slides showed benign-appearing cystic structures in the margins that persisted after it had appeared that the original malignancy had been cleared. After taking another Mohs stage due to clinical suspicion, additional SCC was found and was subsequently cleared after two more Mohs stages. To the best of our knowledge, this is the first report to histologically demonstrate biopsy-proven invasive SCC with benign-appearing cystic structures on frozen sections.

## Introduction

Squamous cell carcinoma (SCC) is the second most common cutaneous malignancy that arises from atypical keratinocytes of the epidermis [1]. Histologically, it can be classified on a spectrum from well to poorly differentiated, can be invasive versus in-situ, and has varying potential for perineural invasion, lymphovascular invasion, and metastasis; however, it is unusual to see cystic structures [1]. Epidermal inclusion cysts (EIC) and milia are typically clinically evident as round structures filled with keratin [1]. Histologically, they are lined by squamous epithelium, a preserved granular layer, and keratinous material in the lumen [1]. Rarely, these benign neoplasms can transform into a malignancy [1]. We report a case of biopsy-proven, moderately well-differentiated invasive SCC treated with Mohs surgery that showed benign appearing milia/cyst-like structures in the margins.

## Case presentation

**Figure 1:**
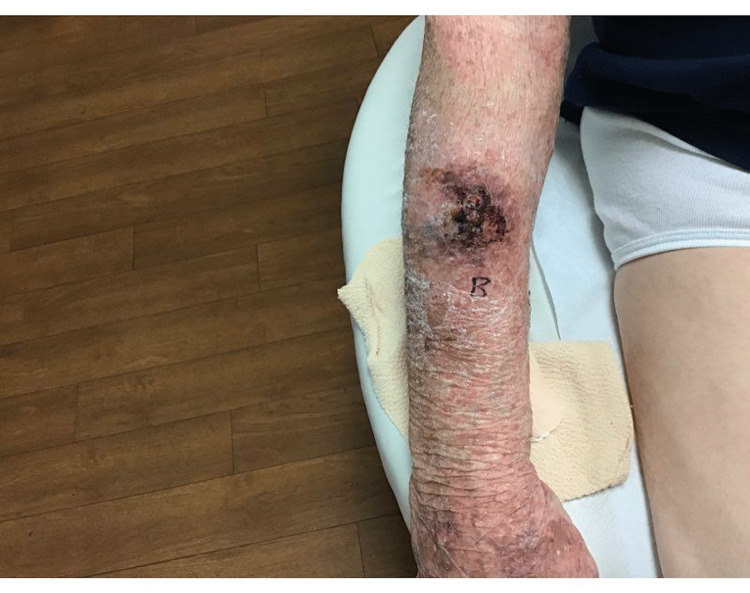
SCC right (R) forearm A 2.6 x 2.3 cm scaly nodule on the right dorsal forearm. A shave biopsy revealed invasive well-differentiated SCC. SCC: Squamous cell carcinoma

A 91-year-old man with a previous medical history of a cerebrovascular accident and basal cell carcinoma of the chin (status post Mohs surgery in 2018) presented to the clinic for a one-month history of an enlarging, painful, draining nodule on the right forearm. The patient had no prior history of a chronic wound in the area, trauma, or osteomyelitis. The physical exam was significant for a 2.6 x 2.3 cm scaly nodule on the right dorsal forearm (Figure 1).

**Figure 2:**
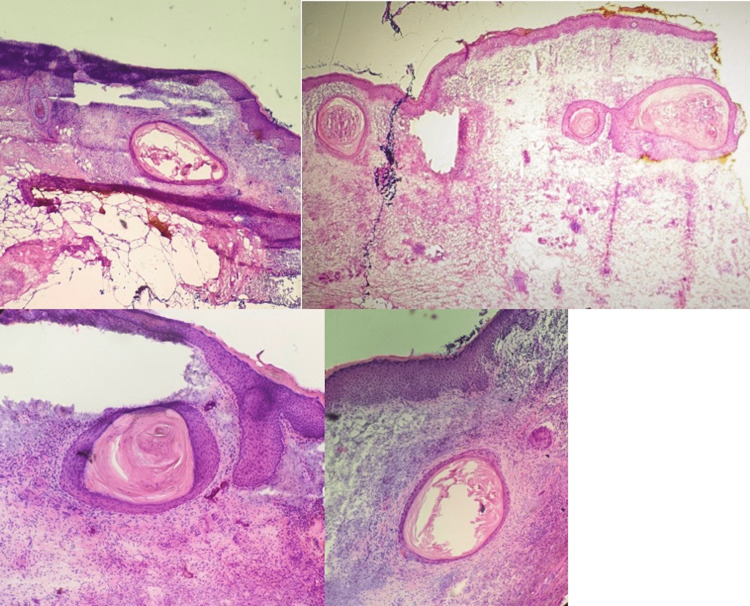
Mohs Stage I frozen sections Sections were positive for full-thickness epidermal keratinocyte atypia with large hyperchromatic and pleomorphic nuclei. Dermal cystic structures are observed. Top left and right: Hematoxylin and eosin (H&E) 4x; Bottom left and right: H&E 10x.

A shave biopsy revealed invasive well-differentiated SCC without definitive perineural, lymphovascular, or subcutaneous invasion. Given the size and ill-defined margins of the lesion, the patient qualified for Mohs surgery. On frozen sections, stage I was positive for full-thickness epidermal keratinocyte atypia with large hyperchromatic and pleomorphic nuclei. In addition, dermal keratin-filled cystic structures lined by stratified squamous epithelium with a preserved granular layer were observed in the margins, histologically emulating EICs versus milia (Figure 2). 

**Figure 3:**
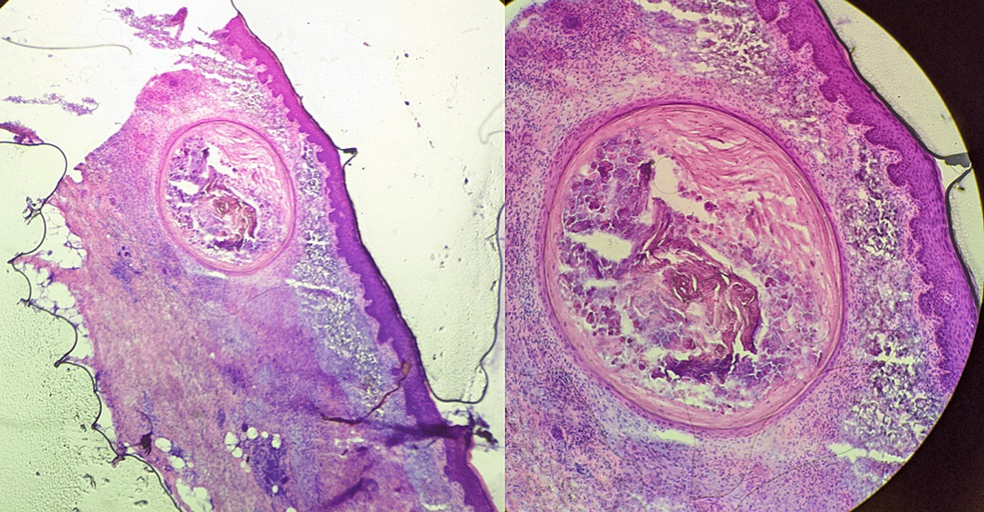
Mohs Stage II frozen sections Persistence of dermal cystic structures without keratinocyte atypia. Left: H&E 4x; Right: H&E 10x.

No malignant squamous proliferation or atypia was seen in the cyst wall or lumen. No perineural or lymphovascular invasion was noted. Stage I tissue was thawed and sent for permanent sections for more accurate staging. Pathology showed moderately well-differentiated squamous cell carcinoma without perineural or lymphovascular invasion. In stage II, no keratinocyte atypia was noted, but the benign-appearing dermal cystic structures in the margins persisted (Figure 3).

**Figure 4:**
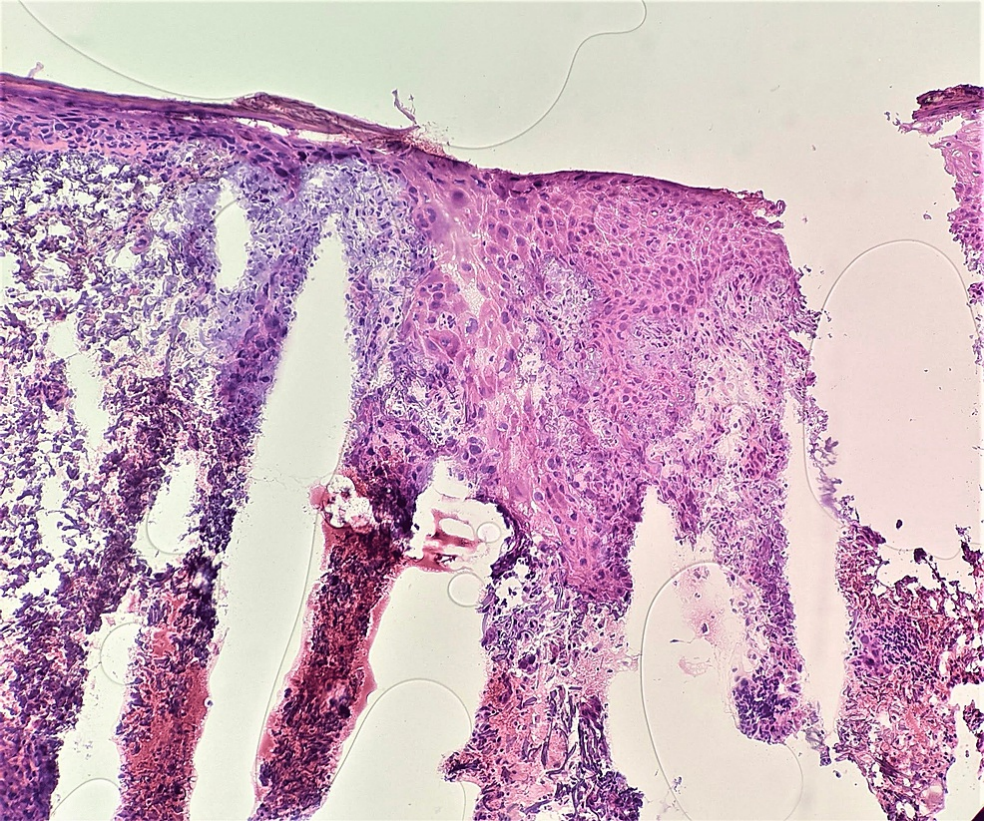
Mohs Stage III frozen sections Renewed areas of keratinocyte atypia as well as finger-like epidermal projections resembling pseudoepitheliomatous hyperplasia. H&E 10x.

Observation versus further Mohs excision was debated. Despite the bland features of the cystic structures, there was not a satisfactory, plausible explanation for them. Due to clinical suspicion, the decision was made to continue surgery. Interestingly, stage III showed renewed areas of keratinocyte atypia projecting from the undersurface of the epidermis and extending into the dermis, suspicious for SCC, as well as finger-like epidermal projections resembling pseudoepitheliomatous hyperplasia (Figure 4).

**Figure 5:**
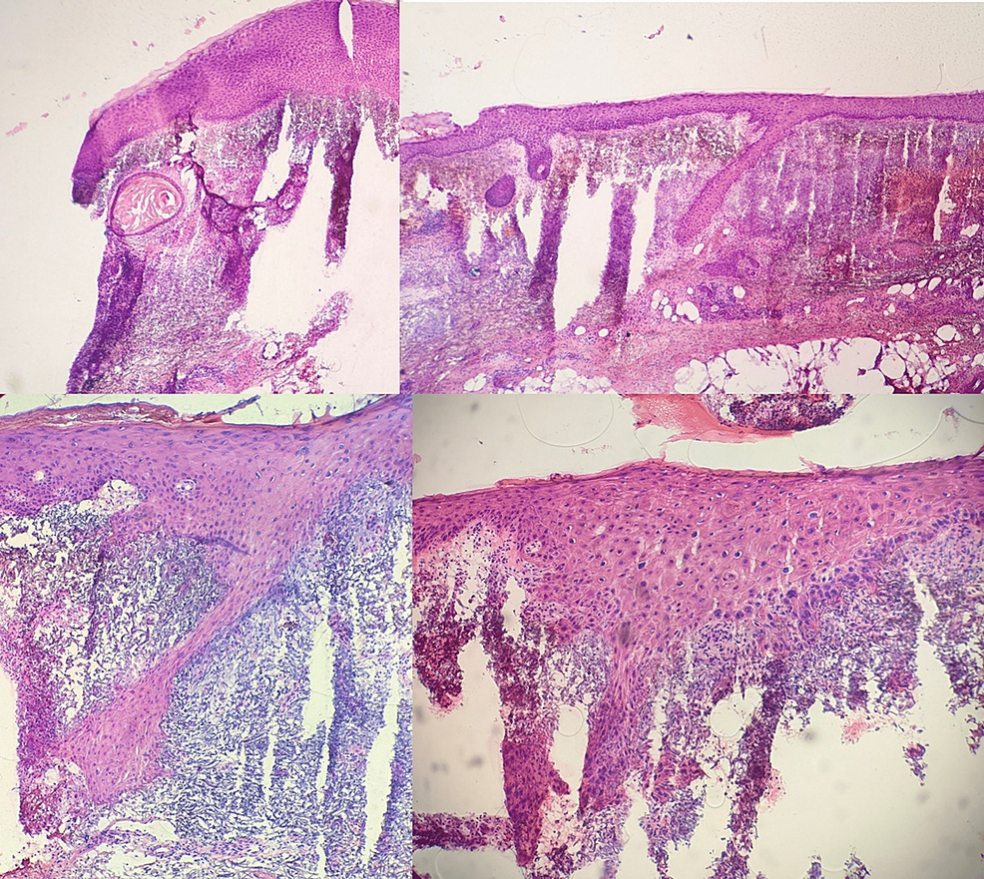
Mohs Stage IV frozen sections Similar findings to Stage III with recurrence of a cystic structure. Top left: cystic structure, H&E 10x; Top right and bottom left: Finger-like pseudoepitheliomatous hyperplasia projections, H&E 10x; Bottom right: Renewed areas of keratinocyte atypia suspicious for squamous cell carcinoma (SCC), H&E 10x.

Stage IV showed similar findings along with the recurrence of a cystic structure (Figure 5).

Stage V showed clear margins and no further cystic structures; the defect was then closed with an intermediate layered linear repair.

## Discussion

Histologically, a milium (classified as a small epidermal cyst) or EIC is lined by squamous epithelium, a preserved granular layer, and epidermal-type keratinous material in the lumen [1]. Multiple malignancies have been reported to arise from EICs, including basal cell carcinoma (BCC), SCC, and Merkel cell carcinoma (MCC) [1]. The most common is well-differentiated SCC involving the head and neck further supported by a literature review from 1976-2021 demonstrating 56 cases. The incidence has been reported to range between 0.011% and 9.2% [1]. In most of these cases, much of the cyst lining and lumen was replaced by a squamous proliferation with malignant features (keratinocyte pleomorphism, mitotic figures, prominent nucleoli) [1]. The cysts on our histology showed no such malignant features. To the best of our knowledge, this is the first report to demonstrate biopsy-proven well-differentiated SCC with the appearance of benign cystic structures on Mohs frozen sections, as opposed to malignant transformation of an EIC/milium. Interestingly, only cystic structures were identified in stage II without the presence of malignant features. However, it was not until stages III and IV that renewed features of keratinocyte atypia were seen in the epidermis and dermis, and in stage IV that recurrence of one cyst-like structure was seen.

A rare subtype of SCC, termed infundibulocystic SCC (reflecting true infundibular differentiation of a follicular tumor), was described in 2008 by Kossard et al., in which multiple pleomorphic micro-or dilated cysts infiltrated into the deep dermis and the subcutaneous tissue [2]. The cysts contained laminated or compact keratin. Misago et al. described eight further such cases in 2011, with only one more similar case report thereafter [3,4]. Our case, being not only rare, was distinct in that no cystic structures were seen in the initial biopsy; it was not classified as an infundibulocystic SCC. Rather, these benign-appearing cysts first appeared upon initiation of Mohs surgery and persisted after all other malignant cells were cleared. The surgeon (JSR), had prior experience with cystic structures in the margins of keratoacanthomas (KA), and has previously strived for their clearance, despite their benign appearance. Two reports in the literature describe cysts on the histology of an early-stage KA, which arises from the pilosebaceous unit [5,6].

Cystic structures have been seen in BCCs, where they tend to be scattered throughout the anastomosing basaloid tumor islands. A controversial infundibulocystic variant has been described, accounting for less than 5% of all BCCs [7-12]. It is more common in basal cell nevoid syndrome and results from a mutation in the Hedgehog pathway [13]. It tends to be superficial, small, well-differentiated, and lacking in high-grade cytologic atypia [8]. This variant may clinically resemble an EIC more than the traditional “pearly, translucent, rolled borders” appearance [7]. The mechanism of cyst formation has been theorized to be due to massive cell necrosis from rapid tumor growth [7]. MCC has also been reported uncommonly to show significant microcystic spaces on histology [14]. Other neoplasms that have been described with cystic histologic features include basaloid follicular hamartoma, trichoblastoma, and trichoepithelioma [15].

## Conclusions

To the best of our knowledge, this is the first report in the literature to demonstrate and highlight this unique phenomenon. No cystic structures were seen on the initial biopsy of well-differentiated invasive SCC. Frozen sections during Mohs surgery showed benign-appearing cystic structures in the margins that persisted after it appeared that the original malignancy had cleared. After taking another stage due to clinical suspicion, additional SCC was found and was subsequently cleared after two more stages. The senior author’s experience with seeing milia-like cysts in the margins of KAs on frozen sections during Mohs surgery and the unusual presence of multiple cystic structures prompted the decision to excise additional margins despite clearance of the tumor. We encourage Mohs surgeons to maintain a healthy level of suspicion for any seemingly benign cystic structures that appear on frozen sections of KAs/SCCs. We would recommend considering further excision until these structures, along with the original malignancy, are clear.
